# Reversal of memory and autism-related phenotypes in *Tsc2* ^*+/−*^ mice via inhibition of *Nlgn1*

**DOI:** 10.3389/fcell.2023.1205112

**Published:** 2023-05-24

**Authors:** Kleanthi Chalkiadaki, Elpida Statoulla, Maria Zafeiri, Nabila Haji, Jean-Claude Lacaille, Craig M. Powell, Seyed Mehdi Jafarnejad, Arkady Khoutorsky, Christos G. Gkogkas

**Affiliations:** ^1^ Biomedical Research Institute, Foundation for Research and Technology Hellas, Ioannina, Greece; ^2^ Centre for Discovery Brain Sciences, University of Edinburgh, Edinburgh, Scotland, United Kingdom; ^3^ Department of Neurosciences, Center for Interdisciplinary Research on Brain and Learning (CIRCA), Research Group on Neural Signaling and Circuitry (GRSNC), Université de Montréal, Montreal, QC, Canada; ^4^ Department of Neurobiology, Civitan International Research Center at UAB Heersink School of Medicine, University of Alabama at Birmingham Heersink School of Medicine, Birmingham, AL, United States; ^5^ Patrick G. Johnston Centre for Cancer Research, Queen’s University Belfast, Belfast, Northern Ireland, United Kingdom; ^6^ Department of Anesthesia, Faculty of Dental Medicine and Oral Health Sciences, McGill University, Montréal, QC, Canada

**Keywords:** tuberous sclerosis, mTOR, translational control, autism, memory

## Abstract

Tuberous sclerosis complex (TSC) is a rare monogenic disorder co-diagnosed with high rates of autism and is caused by loss of function mutations in the TSC1 or TSC2 genes. A key pathway hyperactivated in TSC is the mammalian/mechanistic target of rapamycin complex 1 (mTORC1), which regulates cap-dependent mRNA translation. We previously demonstrated that exaggerated cap-dependent translation leads to autism-related phenotypes and increased mRNA translation and protein expression of Neuroligin 1 (Nlgn1) in mice. Inhibition of Nlgn1 expression reversed social behavior deficits in mice with increased cap-dependent translation. Herein, we report elevated translation of *Nlgn1* mRNA and an increase in its protein expression. Genetic or pharmacological inhibition of Nlgn1 expression in *Tsc2* ^*+/−*^ mice rescued impaired hippocampal mGluR-LTD, contextual discrimination and social behavior deficits in *Tsc2* ^*+/−*^ mice, without correcting mTORC1 hyperactivation. Thus, we demonstrate that reduction of Nlgn1 expression in *Tsc2* ^*+/−*^ mice is a new therapeutic strategy for TSC and potentially other neurodevelopmental disorders.

## 1 Introduction

### 1.1 Tuberous sclerosis and autism spectrum disorder

Loss of function mutations in the genes encoding tuberous sclerosis proteins TSC1 (hamartin) and TSC2 (tuberin) lead to the tuberous sclerosis complex (TSC), a rare autosomal dominant genetic disorder, with very high penetrance (Touraine et al., 2022). TSC manifests with multi-organ pathologies such as benign-tumor growth, skin abnormalities, lung lesions, and kidney problems. Central to TSC pathology are pervasive neurological symptoms such as seizures, cognitive difficulties, changes in behavior such as aggressive behavior, self-harming, attention deficit hyperactivity disorder and autism spectrum disorder (ASD) in ∼50% of children diagnosed with TSC.

ASD is a polygenic condition (Vorstman et al., 2017), affecting distinct domains of behavior and is accompanied by altered brain connectivity and excitation/inhibition dysregulation, which may engender a plethora of comorbid conditions such as epilepsy, sleep disturbance, anxiety, depression and peripheral changes in feeding and gastrointestinal function (Doernberg and Hollander, 2016). Several monogenic disorders, including TSC, are caused by mutations in genes (*TSC1/TSC2*, *PTEN*; phosphatase and tensin homolog, *NF1*; neurofibromin 1, *FMR1*; fragile X messenger ribonucleoprotein 1), which encode proteins that participate in signaling cascades converging to the mammalian/mechanistic target of rapamycin complex 1 (mTORC1) pathway (Kelleher and Bear, 2008), providing a possible unifying mechanistic explanation for several forms of syndromic and/or idiopathic ASD. TSC1/2 inhibit the small GTPase Rheb (RAS homolog-mTORC1 binding), which activates mTORC1 (Garami et al., 2003). Thus, loss of function of TSC1/2 engenders mTORC1 hyperactivation.

### 1.2 mTOR and regulation of mRNA translation

mTORC1 integrates extrinsic and intrinsic cues to elicit multilevel cellular responses ranging from cell proliferation and modulation of gene expression to autophagy and apoptosis, primarily by recruiting multiprotein signaling cascades (Liu and Sabatini, 2020). The best ascribed function of mTORC1 signaling is regulation of mRNA translation via phosphorylation of two major downstream effectors: 4E-BPs (eukaryotic initiation factor 4E-binding proteins 1, 2 and 3) and S6Ks (ribosomal protein S6 kinases 1 and 2), which regulate cap-dependent initiation of translation (Mamane et al., 2006). Hypo-phosphorylated 4E-BPs bind to the cap-binding protein, the eukaryotic translation initiation factor 4E (eIF4E), to preclude eIF4F complex formation, an early step in translation initiation (Sonenberg, 2008). Upon phosphorylation of 4E-BPs by mTORC1, eIF4F complex formation and cap-dependent translation are stimulated. Cap-dependent translation preferentially promotes the synthesis of a subset of mRNAs termed “eIF4E-senstitive” (Hinnebusch et al., 2016).

### 1.3 Study rationale

We previously showed that deletion of the predominant mouse brain isoform 4E-BP2, mimicking hyperactivation of the mTOR/4E-BP/eIF4E axis, engenders behaviors reminiscent of ASD (social behavior and repetitive/stereotypic behaviors), accompanied by hippocampal excitation/inhibition imbalance (Gkogkas et al., 2013). This phenotype was explained by preferential exaggerated cap-dependent translation of synaptic mRNAs such as neuroligin 1 (*Nlgn1*), by eIF4E, in the absence of 4E-BP2. Overexpression of eIF4E induces similar behavioral and molecular/cellular phenotypes in mice (Santini et al., 2013). Similarly, mTOR/4E-BP hyperactivation in *Tsc2* ^*+/−*^ mice is accompanied by a wide range of rapamycin-sensitive phenotypes: decreased hippocampal metabotropic glutamate receptor long-term depression (mGluR-LTD) (Auerbach et al., 2011), deficits in learning and memory (Ehninger et al., 2008) and autism-related behaviors such as changes in social interaction (Sato et al., 2012).

Nlgns are cell-adhesion molecules that regulate synaptic structure function, with known mutations linked to the pathogenesis of autism (Trobiani et al., 2020). Interestingly *Nlgn1* knockout (KO) mice display modest changes in social behavior, but a profound increase in repetitive, stereotyped grooming and deficits in spatial learning and memory (Blundell et al., 2010). On the other hand, overexpression of Nlgn1 leads to learning and synaptic plasticity deficits by altering the E/I balance in the hippocampus (Dahlhaus et al., 2010).

Herein, we show that translation of *Nlgn1* mRNA is significantly increased in the hippocampus of *Tsc2* ^*+/−*^ mice, leading to increased *Nlgn1* protein expression. Pharmacological inhibition of mTORC1 or of cap-dependent translation restored translation of *Nlgn1* mRNA in *Tsc2* ^*+/−*^ mice. Genetic deletion of one copy of *Nlgn1* was sufficient to reverse the impairment of mGluR-LTD in *Tsc2* ^*+/−*^ mice. Furthermore, normalizing *Nlgn1* expression in *Tsc2* ^*+/−*^ mice via genetic deletion of one *Nlgn1* gene copy or via treatment with the cap-dependent translation inhibitor 4EGI-1, reversed hippocampal contextual memory impairment and social behavior deficits, without restoring mTORC1 signaling. Altogether, we reveal rescue of autism-related phenotypes in a mouse model of TSC by targeting *Nlgn1*, a key downstream effector molecule regulated by the mTORC1/4E-BP axis.

## 2 Materials and methods

### 2.1 Transgenic mice

All procedures followed the UK Home Office and Canadian Council on Animal Care guidelines and were respectively approved by the University of Edinburgh, McGill University and Université de Montréal. *Tsc2* ^*+/−*^ mice (B6; 129S4-Tsc2tm1Djk/J, Jackson Laboratories) and *Nlgn1* ^*+/−*^ mice (Prof. C. Powell, UT Southwestern) were maintained on the C57Bl/6J background (backcrossed for more than 10 generations). For all experiments, littermates from heterozygote crossings were used both for *Tsc2* and *Nlgn1* models. Male and female mice were used where specified. Food and water were provided *ad libitum*, and mice were kept on a 12 h light/dark cycle. Pups were kept with their dams until weaning at postnatal day 21. After weaning, mice were group housed (maximum of four per cage) by sex. Cages were maintained in ventilated racks in temperature (20–21°C) and humidity (∼55%) controlled rooms, on a 12-h circadian cycle (7a.m.-7p.m. light period). For all behavioral assays, mice were handled/habituated for three consecutive days prior to experimental testing.

### 2.2 Polysome profiling

Polysome profile analysis was carried out as described previously (Gkogkas et al., 2013). Intact hippocampi from 2-month-old male mice were washed with ice-cold PBS containing 100 μg/mL cycloheximide, lysed in a hypotonic lysis buffer (5 mM Tris-HCl pH 7.5, 2.5 mM MgCl_2_, 1.5 mM KCl, 100 μg/mL cycloheximide, 2 mM DTT, 0.5% Triton X-100, and 0.5% sodium deoxycholate) and analyzed using polysome profiling. The Polysome to monosome ratio was calculated as the area under the A254 absorbance curve, using the function describing the absorbance values, processed with the definite integral command in MATLAB. Statistical analysis was carried out with a Student’s t-test.

### 2.3 RT-qPCR

Polysomal or total RNA was analyzed using a Biorad iQ SYBR Green Supermix kit as previously described (Gkogkas et al., 2013). Light polysomes correspond to fractions 11–13 (including monosomes, disomes, trisomes), while heavy polysomes (>3 ribosomes) correspond to fractions 14–18. For all experiments *n* = 4; results are presented in arbitrary units as relative amounts using serial dilutions of cortical or hippocampal RNA as RT-qPCR concentration standards. Statistical analysis was carried out with a Student’s t-test.

### 2.4 Western blotting and antibodies

All tissues were dissociated in RIPA buffer (unless otherwise specified). Western blotting was previously described (Gkogkas et al., 2013). Antibodies against indicated proteins are summarized in Supplementary Table S1. Quantification of immunoblots was performed using ImageJ (NIH). Values were normalized to *β*-tubulin, HSC70, or another control (e.g., for phosphorylation) where specified and presented as percentage or fold change relative to control. Statistical analysis was carried out using a Student’s t-test or One-way ANOVA with a Bonferroni’s post-hoc test, where specified.

### 2.5 *Nlgn1* immunofluorescence and imaging

2-month-old mice were perfused intracardiacally with 4% paraformaldehyde. The brains were postfixed in PFA 4% for 24 h following extraction. The fixed brains were sectioned using a vibratome to acquire 40 µm-thick sections. Sections were permeabilized in 0.3% Triton X-100 in PBS for 30 min, blocked in 5% goat serum for 1 h, incubated with primary antibodies [1:129211, neuroligin (Synaptic Systems)] at 4°C overnight, washed in PBS, incubated with Alexa Fluor 488 or 594 (1:400, Molecular Probes), washed with PBS and mounted on microscope coverslips (Light Diagnostics). The specificity of the antibody was tested and confirmed using neuroligin 1 knockout hippocampal sections. Images were acquired with a Zeiss confocal microscope using a ×63 oil-immersion objective.

### 2.6 LTD field recordings

Transverse hippocampal slices (400 μm), prepared from 30 to 35-day-old male mice, were allowed to recover submerged for at least 2 h at 32°C in oxygenated artificial cerebrospinal fluid (ACSF) containing 124 mM NaCl, 5 mM KCl, 1.25 mM NaH_2_PO_4_, 2 mM MgSO_4_, 2 mM CaCl_2_, 26 mM NaHCO_3_ and 10 mM glucose, and for additional 30 min submerged in a recording chamber at 27°C–28°C, while continuously perfused with ACSF. CA1 and CA3 hippocampal regions were disconnected by a surgical cut. Field extracellular postsynaptic potentials (fEPSPs) were recorded in CA1 stratum radiatum with glass electrodes (2–3 MΩ) filled with ASCF. Schaffer collateral fEPSPs were evoked by stimulation with a concentric bipolar tungsten stimulating electrode placed in mid-stratum radiatum proximal to CA3 region. Baseline stimulation was applied at 0.033 Hz by delivering 0.1 ms pulses, with intensity adjusted to evoke 60% of maximal fEPSPs. Group I metabotropic glutamate receptor (mGluR) agonist (S)-3,5-dihydroxyphenylglycine (DHPG, 50 μM, Tocris) was added to ACSF for 10 min to induce mGluR-mediated LTD. fEPSP slope measurements were performed on digitized analogue recordings using the Clampfit analyze function and between 10% and 90% of maximal fEPSP amplitude. Statistical analyses were done on the 80–90 min period post-LTD induction, using One-way ANOVA, followed by Bonferroni’s post-hoc test.

### 2.7 Context discrimination task

This test was performed as in (Auerbach et al., 2011) and (Ehninger et al., 2008) with modifications. After handling, 2-month-old male mice were allowed to explore context A for 3 min, then conditioned to the training context A (metal floor, black walls, white lighting, no odor) with one 0.8 mA foot shock (2 s duration). Mice were removed 15 s after the shock was given and returned to home cage. Freezing was assessed 24 h later for 3 min as a proxy for contextual fear conditioning. To determine context specificity of the freezing response, mice trained in context A were separated into two groups: 50% of the mice were tested in context A (familiar context) and 50% in context B (novel context; plastic floor, black-white pattern walls, red light, no odor). Statistical analysis was carried out with One-way ANOVA with Bonferroni’s post-hoc test.

### 2.8 Reciprocal social interaction test

Pairs of 3–4 month-old male and female mice were tested for 15 min (testing only same sex and genotype pairs) in an open-field apparatus. Social behavior was analyzed for active interaction (sniffing, allo-grooming, mounting, or following) and rearing behavior. Statistical analysis was carried out using One-way ANOVA with Bonferroni’s post-hoc test.

### 2.9 Tube co-occupancy test (TCOT)

Same-sex mouse dyads were introduced into an arena with Plexiglas walls that were opaque, measuring 39 cm in width, 26 cm in length, and 12 cm in height. The arena was placed 105 cm above ground level on a glass shelf to create a visual cliff, and it was illuminated with a 250-W LED light, providing an approximate of 3,000 lux. Within each open-field box, a single opaque PVC cylinder was present, measuring 7.5 cm in length and 3 cm in diameter, allowing only for joint proximity of the two animals (huddling). The time of tube co-occupancy, single occupancy or vacancy were measured over 180 min.

### 2.10 Intrahippocampal infusion in mice

3–4-month-old mice were deeply anaesthetized with 1 mL kg^-−1^ of body weight of ketamine (55 mg per kg), xylazine (3 mg per kg) and medetomidine hydrochloride (0.3 mg per kg) injected intraperitoneally. Mice were placed in a Kopf stereotactic frame. A midline scalp incision was made to expose the skull, and bregma was used as the stereotaxic zero point. The guide cannulae, each 10 mm in length and 26-gauge in size, were implanted bilaterally into the hippocampi at AP -1.5 mm, ML ±1.65 mm, DV -1.8 mm (Paxinos Mouse Atlas). Cannulae were then fixed onto the skull with dental cement. Dummy cannulae were used to maintain clearance through each guide cannula. Mice were given a recovery period of at least 7 days following the surgery. During the infusion, a needle (33-gauge) was inserted into the guide cannula, 1.0 mm below the cannula tip. Using a peristaltic pump (connected to Hamilton 10 μL microsyringe via PE tubing), 0.5 μL per side of vehicle (50% DMSO in 100 mM Tris-Cl pH 7.2) rapamycin (Sigma, R0395, 30 nM per side) or 4EGI-1 (Sigma, 324519, 20 μg/μL) were infused into each hippocampus at a rate of 0.1 μL/min for 5 min. To prevent reflux, infusion needles remained in the guide cannulae for an additional 5 min following infusion.

### 2.11 Preparation of synaptososomes

Synaptosomes were prepared from freshly dissected hippocampus from 3–4-month-old mice as described in (Simbriger et al., 2020). Briefly, following rapid dissection, hippocampi were homogenized using a glass Dounce homogenizer in ice–cold sucrose buffer (320 mM sucrose, 5 mM Tris-HCl pH 7.4, 1 mM EDTA), and then centrifuged for 10 min at 1000 x *g*, 4°C. The pellet was resuspended in the same sucrose buffer and centrifuged again as before. The combined supernatant from both spins was further centrifuged for 10 min at 21,000 x *g*, 4°C, to pellet crude synaptosomes.

### 2.12 Statistical analysis

Experimenters were blinded to animal genotype during testing and scoring. All data are presented as mean ± S.E.M. (error bars). Statistical significance was set *a priori* at 0.05 (n.s.: non-significant). No nested data were collected in this study; n number corresponds to single observations (biological replicates). No randomization was performed in drug treatment. Sample size for behavioral, electrophysiological and biochemical analyses was determined with power calculations, based on previous data and effect size in refs (Gkogkas et al., 2013; Tuttle et al., 2017) using G*Power (a = 0.05, b: 0.2) (Faul et al., 2009). Normality test was performed using the Shapiro-Wilk test (α = 0.05). Details for statistical analysis are provided in Supplementary Table S2.

## 3 Results

### 3.1 Exaggerated mTORC1/cap-dependent translation of Nlgn1 downstream of Tsc2

**FIGURE 1:**
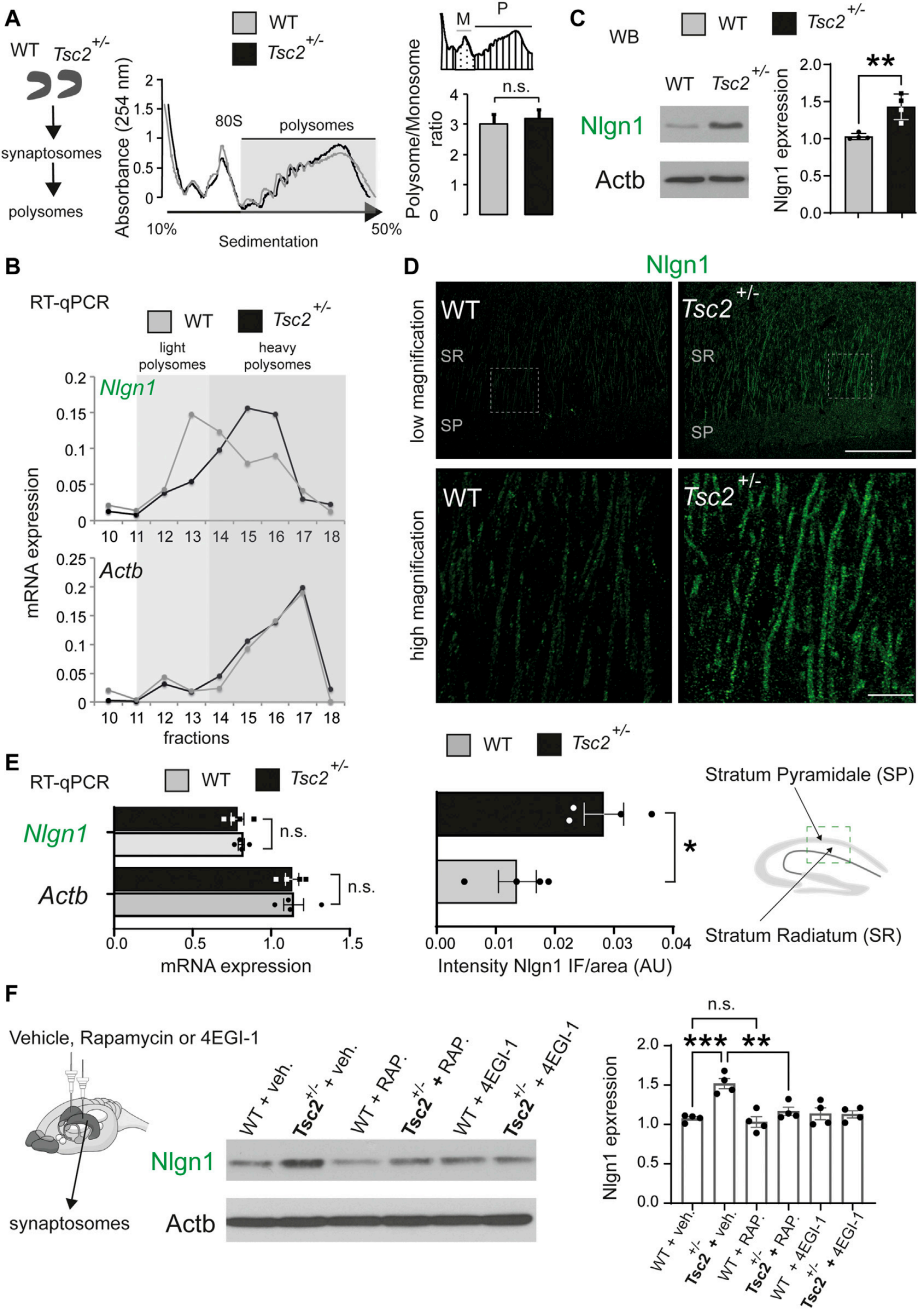
Exaggerated translation of *Nlgn1* mRNA in *Tsc2* ^*+/−*^ mice. **(A)** Polysome fractionation in synaptosomes prepared from dissected hippocampi. Left: No difference in overall polysome sedimentation profiles between wild type (WT) and *Tsc2* ^*+/−*^ mice. Positions of the 80S ribosome peak and polysomes are indicated. Right: No difference in polysome (P)/monosome (M) ratio between WT and *Tsc2* ^*+/−*^ mice (*n* = 3, *p* = 0.15, Student’s t-test). **(B)** Increased *Nlgn1* mRNA amounts associated with heavy polysome fractions in hippocampal synaptosomes from *Tsc2* ^+/−^ mice. RT-qPCR analysis of RNA extracted from polysomal fractions with specific primers for *Nlgn1.* No change in *Actb* mRNA distribution (*n* = 3; *p* = 0.003 light/heavy polysomes Student’s t-test). **(C)** Increased Nlgn1 protein amounts in synaptosomes from hippocampal lysates in *Tsc2* ^+/−^ mice. (*n* = 4, **p < 0.01, Student’s t-test). **(D)** Increased Nlgn1 immunostaining in CA1 pyramidal cells in *Tsc2* ^*+/−*^ hippocampus compared to WT shown with immunofluorescence. Top: low magnification; bottom: high magnification; SR, Stratum Radiatum; SP,: Stratum Pyramidale (*n* = 4, *p < 0.05, Student’s t-test). Scale bars: low magnification 80 μm, high magnification 20 μm. **(E)** No changes in *Nlgn1* mRNA levels in *Tsc2* ^*+/−*^ mice. RT-qPCR analysis of total *Nlgn1* or *Actb* mRNA in hippocampal synaptosomes from WT or *Tsc2* ^*+/−*^ mice (*n* = 4, Student’s t-test). **(F)** Chronic sub-threshold intrahippocampal infusion of rapamycin (30 nM) or 4EGI-1 (20 μg/μL) reduced Nlgn1 protein expression in synaptosomes from *Tsc2* ^*+/−*^ mice hippocampi. Left: Representative immunoblots of hippocampal synaptosome lysate are shown, probed with antisera against the indicated proteins; Actb: loading control. Right: Quantification of Nlgn1 protein expression in immunoblotting for the different groups (One-way ANOVA, with Bonferroni’s post-hoc, *n* = 4 per group, **p < 0.01, ***p < 0.001).

We previously demonstrated that *Nlgn* mRNAs are preferentially translated downstream of 4E-BP2/eIF4E and showed that knockdown of *Nlgn1* in the hippocampus rescued behavioral deficits in *Eif4ebp2* ^*−/−*^ mice (Gkogkas et al., 2013). Since in Tuberous Sclerosis there is hyperactivation of mTORC1 and subsequently hyperphosphorylation of its downstream effectors, 4E-BPs and S6Ks, we reasoned that *Nlgn1* mRNA translation might be increased in *Tsc2* ^*+/−*^ hippocampi. To examine translation of *Nlgn1*, we carried out polysome profiling on synaptosomes purified from dissected hippocampi of *Tsc2* ^*+/−*^ and wild type (*Tsc2* ^*+/+*^; WT) mice. We did not detect any significant differences between *Tsc2* ^*+/−*^ and wild type groups in the overall sedimentation profiles of hippocampal tissue (Figure 1A), as evidenced by the unchanged Polysome/Monosome ratio (Figure 1A). RT-qPCR analysis of the RNA extracted from sucrose density gradient fractions revealed that *Nlgn1* mRNA associated with heavier polysome fractions in *Tsc2* ^*+/−*^ synaptosomes compared with wild type (Figure 1B). In accordance with this finding, Nlgn1 protein expression was significantly increased (∼40%) in *Tsc2* ^*+/−*^ synaptosomes compared with wild type (Figure 1C), and in Nlgn1 immunofluorescence staining of the hippocampus stratum radiatum in *Tsc2* ^*+/−*^ mice (Figure 1D). We did not observe significant changes in total *Nlgn1* mRNA amounts between the different genotypes (Figure 1E). Together, these findings indicate that the increased *Nlgn1* expression in *Tsc2* ^*+/−*^ is due to translational and not transcriptional changes*.* To establish a direct link between translational control via mTORC1 hyperactivation and *Nlgn1* protein expression in *Tsc2* ^*+/−*^ brain, we performed chronic *in vivo* treatment of *Tsc2* ^*+/−*^ mice with rapamycin (mTORC1 inhibitor) or 4EGI-1 (inhibitor of cap-dependent translation that disrupts the interaction between eIF4E and eIF4G). We previously established subthreshold dosage of rapamycin and 4EGI-1 that have no effect on *Nlgn1* expression or autism-related and learning/memory phenotypes in wild type mice (Gkogkas et al., 2013). A regimen of 5 days of intrahippocampal rapamycin (30 nM) or 4EGI-1 (20 μg/μL) administration significantly reduced Nlgn1 protein amounts in *Tsc2* ^*+/−*^ but not in WT hippocampal synaptosomes (Figure 1F). Altogether, hyperactivation of mTORC1 in *Tsc2* ^*+/−*^ mice stimulates cap-dependent translation of *Nlgn1* mRNA, resulting in increased *Nlgn1* protein expression.

### 3.2 Reversal of *Tsc2* ^*+/−*^related phenotypes via *Nlgn1* deletion

**FIGURE 2:**
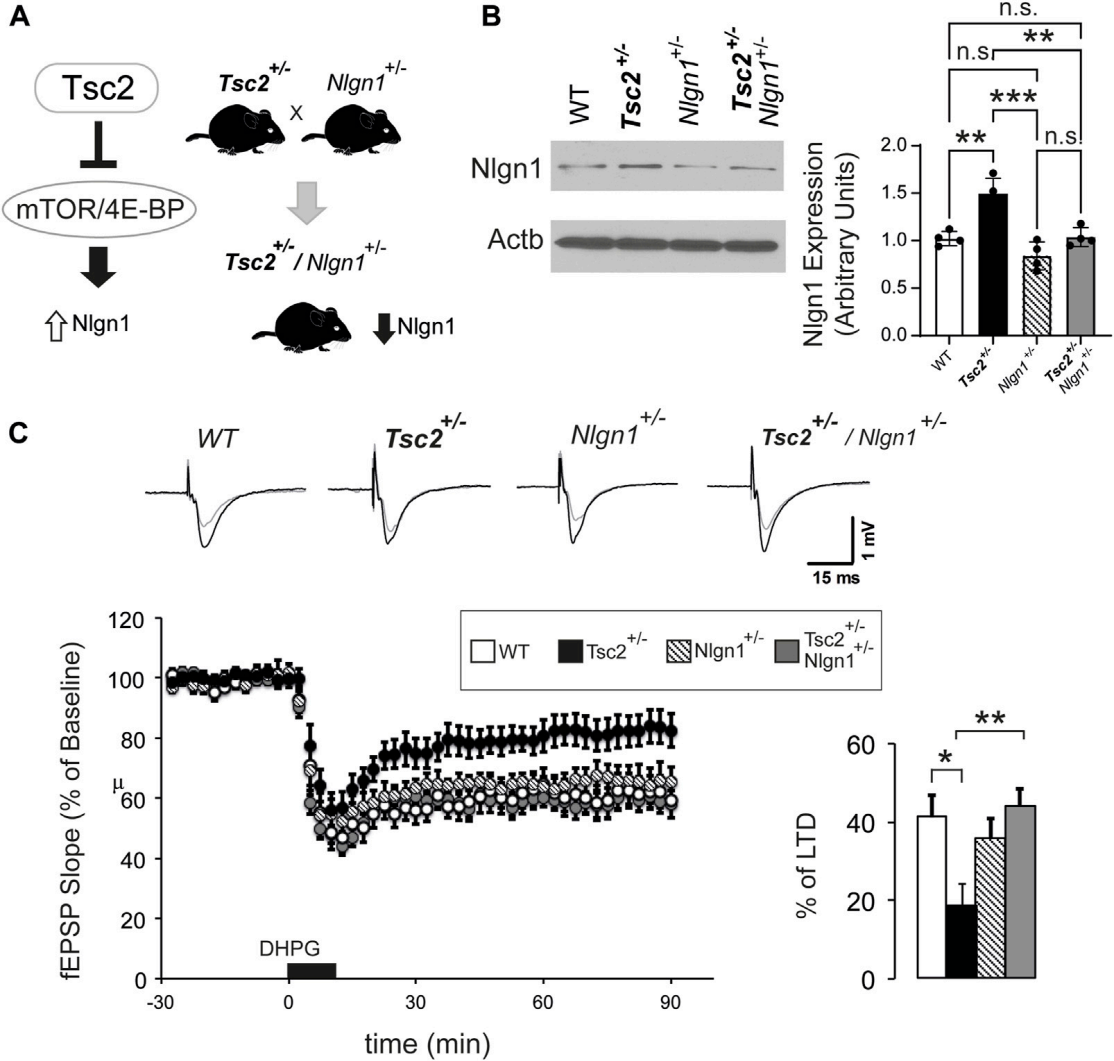
Nlgn1 haploinsufficiency rescues aberrant mGluR-LTD in *Tsc2* ^*+/−*^ mice. **(A)** Outline of breeding strategy to reduce Nlgn1 expression in *Tsc2* ^*+/−*^ mice. **(B)** *Nlgn1* haploinsufficiency restores Nlgn1 expression in synaptosomes prepared from *Tsc2* ^*+/−*^ hippocampi. Representative immunoblots of hippocampal synaptosome lysates probed with antisera against the indicated proteins; Actb: loading control. Quantification of Nlgn1 protein expression is shown (One-way ANOVA, with Bonferroni’s post-hoc, *n* = 4 per group, **p < 0.01, ***p < 0.001). **(C)** *Nlgn1* deletion rescued mGluR-LTD deficits in *Tsc2* ^*+/−*^ mice. *Top:* Representative traces from 4 groups (baseline in black and recording at 90 min in grey). Bottom left: LTD was induced by application of DHPG (50 µM for 10 min). Field excitatory postsynaptic potentials (fEPSP) were recorded over a 90-min period after DHPG-induced LTD. Bottom right: Quantification of the fEPSP slope (% decrease from baseline) during the last 10 min of the recording. One-way ANOVA with Bonferroni’s post-hoc n for groups: WT (13), *Tsc2* ^*+/−*^ (12), *Nlgn1* ^*+/−*^ (9) and *Tsc2* ^*+/−*^/*Nlgn1* ^*+/−*^ (12), *p < 0.05, **p < 0.01.

Previously, we showed that lentivirus-mediated knockdown of *Nlgn1* reversed synaptic and behavioral deficits of *Eif4ebp2* ^*−/−*^ mice (Gkogkas et al., 2013). Given the increased *Nlgn1* expression in the hippocampus of *Tsc2* ^+/−^ mice, we hypothesized that reducing *Nlgn1* protein amounts could rescue the reduced mGluR-LTD in *Tsc2* ^*+/−*^ mice (Auerbach et al., 2011). To achieve a reduction of *Nlgn1* amounts equivalent to our previous findings (Gkogkas et al., 2013), we first deleted one copy of *Nlgn1 in Tsc2* ^+/−^ mice. We crossed *Nlgn1* ^*+/−*^ to *Tsc2* ^*+/−*^ to obtain *Tsc2* ^*+/−*^ */Nlgn1* ^*+/−*^ mice (Figure 2A) and confirmed that deletion of one *Nlgn1* allele reduced *Nlgn1* protein amounts in the hippocampus to WT levels (Figure 2B). To examine the effect of the normalization of *Nlgn1* expression in *Tsc2* ^*+/−*^ */Nlgn1* ^*+/−*^ mice on hippocampal mGluR-LTD, we induced LTD by stimulating group I metabotropic glutamate receptors in hippocampal slices with DHPG (Figure 2C). *Tsc2* ^*+/−*^ mice showed impaired mGluR-LTD relative to WT mice (Figure 2C). Deletion of one copy of *Nlgn1* was sufficient to rescue the mGluR-LTD impairment in *Tsc2* ^*+/−*^ */Nlgn1* ^*+/−*^ mice compared with WT (Figure 2C).

**FIGURE 3:**
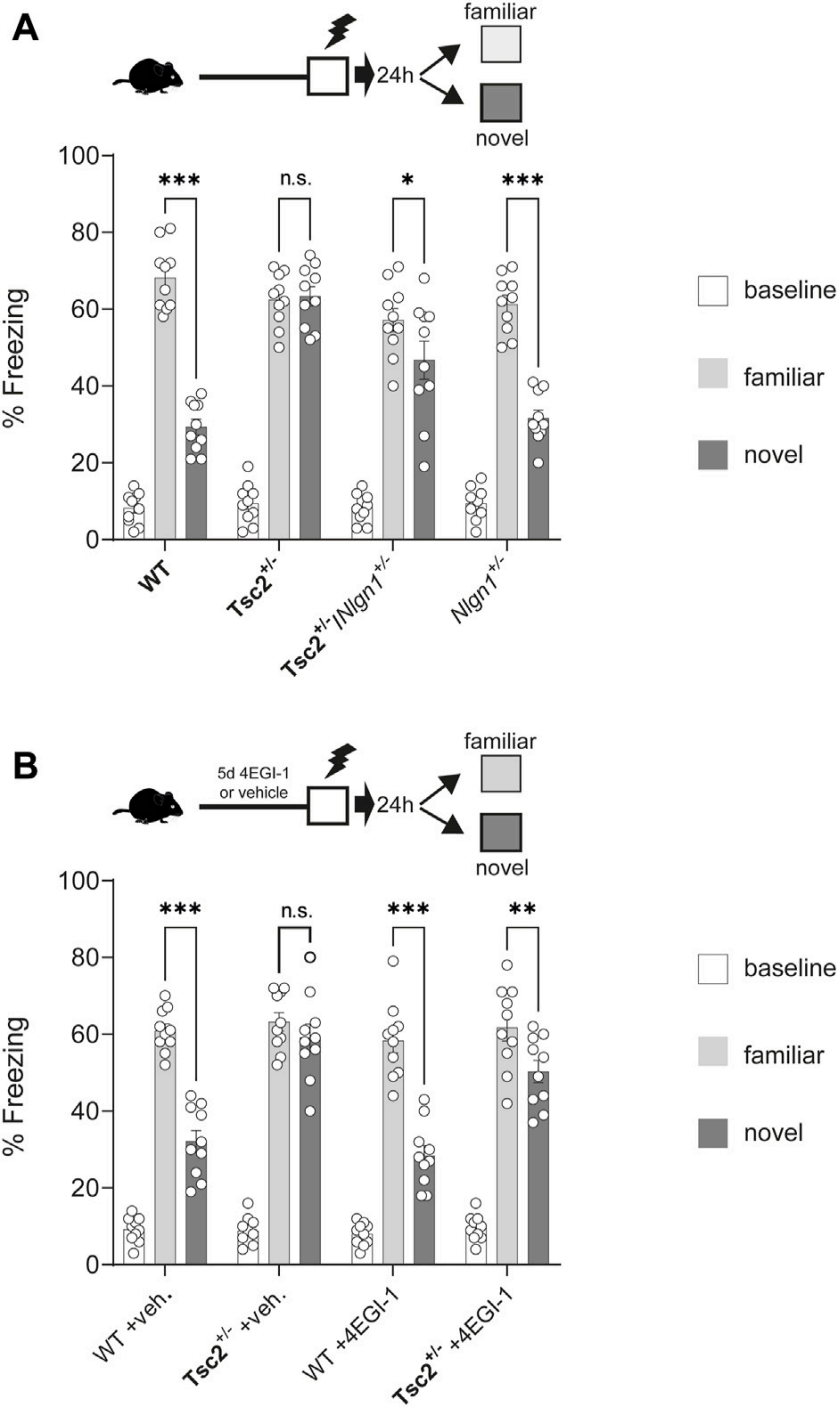
Rescue of hippocampal memory deficits in *Tsc2* ^*+/−*^ mice via *Nlgn1* haploinsufficiency or inhibition of cap-dependent translation with 4EGI-1. *Nlgn1* haploinsufficiency rescued context discrimination deficits in *Tsc2* ^*+/−*^ mice. **(A,B)** Chronic 4EGI-1 treatment rescued context discrimination deficits in *Tsc2* ^*+/−*^ mice. Percentage (%) Freezing shown at baseline, in familiar or novel context for the groups depicted; *n* = 10 for all groups; veh = vehicle. Two-way ANOVA with Bonferroni’s post-hoc *p < 0.05, **p < 0.01, ***p < 0.001.

We next examined whether the correction of synaptic deficits in *Tsc2* ^*+/−*^ via *Nlgn1* deletion could reverse hippocampal-dependent context discrimination, which is impaired in *Tsc2* ^*+/−*^ mice (Ehninger et al., 2008). To this end, mice were trained in Context A (receiving a footshock) (Figure 3). Subsequently, 50% of the animals were tested in Context A (familiar) and the remaining 50% in Context B (novel). *Tsc2* ^*+/−*^ mice failed to discriminate between the two contexts (Figure 3A), as demonstrated by the non-significant difference in freezing percentage between familiar and novel contexts, in accordance with previous reports (Ehninger et al., 2008; Auerbach et al., 2011). Conversely, *Tsc2* ^*+/−*^ */Nlgn1* ^*+/−*^ mice were able to discriminate between the two different contexts (Figure 3A), but without achieving complete rescue to wild type levels. Taken together, these data suggest that deletion of one copy of *Nlgn1*, restoring its protein expression to wild-type levels, is sufficient to reverse synaptic and behavioral deficits in a mouse model of TSC.

To further validate the rescue effect of *Nlgn1* deletion in *Tsc2* ^*+/−*^ mice, we used the 4EGI-1 inhibitor of cap-dependent translation, which we showed is effective in restoring Nlgn1 expression (Figure 1F). Chronic infusion (5 days) of 4EGI-1 (20 μg/μL, for 5 days) in the dorsal hippocampus led to a restoration of context discrimination in *Tsc2* ^*+/−*^ mice, but without achieving complete rescue to wild type levels (Figure 3B). Notably, the subthreshold regimen of 4EGI-1 that we used did not alter context discrimination behavior in WT mice (Figure 3B).

**FIGURE 4:**
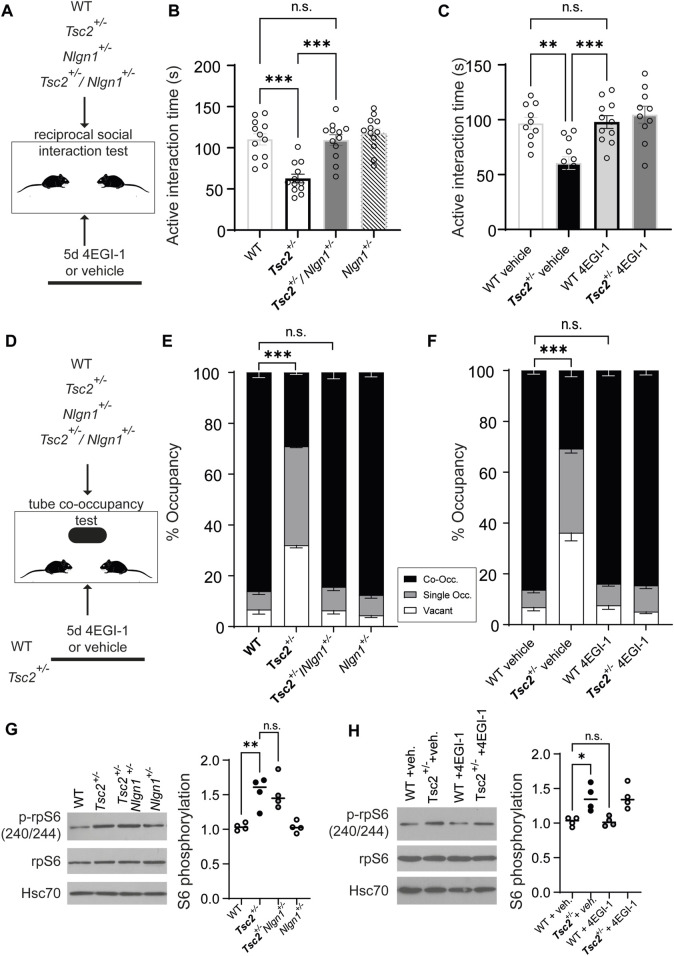
Rescue of social behavior deficits in *Tsc2* ^*+/−*^ mice via *Nlgn1* haploinsufficiency or inhibition of cap-dependent translation with 4EGI-1 but no normalization of mTORC1 signaling. **(A)** Outline of the Reciprocal social interaction test for the indicated groups. **(B,C)** Active interaction during reciprocal social interaction is shown. For A, *n* = 10 for all groups. For **(B)** WT + veh (10), *Tsc2* ^*+/−*^ + veh. (12), WT +4EGI-1 (11), *Tsc2* ^*+/−*^ +4EGI-1 (10); veh = vehicle. **(D)** Outline of the Tube co-occupancy test for the indicated groups. **(E,F)** % Occupancy of the tube in the arena (Co-Occ: co-occupancy, Single Occ.: single occupancy or vacant). **(G)** *Nlgn1* haploinsufficiency or **(H)** 4EGI-1 treatment do not restore mTORC1 signaling in hippocampal synaptosomes from *Tsc2* ^*+/−*^ mice. Left: Representative immunoblots of synaptosomes prepared from hippocampi, probed with antisera against the indicated proteins for the groups shown. Hsc70: loading control. Right: Quantification of phospho-rpS6 expression for the indicated groups. For **(A–C)**: One-way ANOVA, with Bonferroni’s post-hoc and **(D–F**): Two-Way ANOVA with Bonferroni’s post-hoc (*p < 0.05, **p < 0.01, ***p < 0.001).

We finally examined behaviors reminiscent of autism (e.g., changes in social interaction) given their high prevalence in TSC patients. We subjected mice to social behavior analysis using a reciprocal social interaction test (Sato et al., 2012) and the tube co-occupancy test (TCOT) for social propinquity (Tuttle et al., 2017). Deletion of one copy of *Nlgn1* or 4EGI-1 treatment (Figure 4A) in *Tsc2* ^*+/−*^ mice reversed deficits in reciprocal social interaction as evidenced by the active interaction time (Figures 4B, C) and also rescued the reduced co-occupancy time in the TCOT test (Figures 4E, F). Taken together, these data reveal that normalization of *Nlgn1* protein expression rescues not only memory-related impairment, but also deficits in social behavior in *Tsc2* ^*+/−*^ mice.

### 3.3 *Nlgn1* deletion in *Tsc2* ^+/−^ mice does not rescue mTORC1 hyperactivation

We reasoned that behavioral rescue following *Nlgn1* expression normalization in *Tsc2* ^+/−^ mice is related to the hippocampal cellular phenotype observed (rescue of impaired hippocampal mGluR-LTD; Figure 2), yet it was still unclear whether *Nlgn1* deletion affected mTORC1 hyperactivation in *Tsc2* ^*+/−*^. To assess mTORC1 activity, we examined its common readout, the phosphorylation status of ribosomal protein S6 (p-rpS6, Figures 4G, H). Deletion of one copy of *Nlgn1* or chronic 4EGI-1 treatment did not affect p-rpS6 in wild type or vehicle groups, and did not correct increased p-rpS6 in Tsc2^*+/−*^/Nlgn1^*+/−*^ (Figure 4G) and Tsc2^*+/−*^ - 4EGI-1 groups (Figure 4H). Thus, mGluR-LTD and behavioral phenotypes (context discrimination, social deficits) were corrected following normalization of *Nlgn1* protein expression, while mTORC1 hyperactivation persisted.

## 4 Discussion

### 4.1 Relevance to ASD and TSC

ASD is a polygenic disorder and elucidating the contribution of converging signaling pathways will shed fresh light on its pathogenesis mechanisms and thus bolster potential new therapeutics. Dysregulated protein synthesis has emerged as a prominent process implicated in several monogenic forms of ASD and intellectual disability (Chen et al., 2019), as well as in sporadic ASD (Hooshmandi et al., 2020), and is often accompanied by changes in key signaling pathways upstream of translation such as mTOR (Rosina et al., 2019). Of these monogenic ASD forms, several conditions such as fragile X syndrome (FXS) and TSC are also linked to exaggerated global and mRNA-specific protein synthesis (Auerbach et al., 2011). It is evident that inhibitors of signaling upstream of protein synthesis (e.g., rapamycin and rapalogs) have the potential to be used as therapeutics, yet off-target and pervasive side effects cannot be excluded (Chu and Pelletier, 2018). We previously showed that the autism-related pathogenic effects of exaggerated cap-dependent translation on mouse brain physiology and behavior can be reversed by targeting “eIF4E-sensitive” mRNAs such as *Nlgn1* (Gkogkas et al., 2013). Herein, we provide the first example of applying this strategy to rescue phenotypes in an adult mouse model of TSC, a rare genetic disorder, which is also a common monogenic form of ASD.

### 4.2 Relevance to other rescue studies in ASD and TSC

Synaptic protein synthesis was previously implicated in neurodevelopmental disorders such as ASD (Louros and Osterweil, 2016). The advent of genome-wide translational profiling (Ingolia et al., 2009) allowed to uncover potential new candidates for therapeutics, whose expression is regulated at the translational level, such as *Nlgn1*. Given the role of Nlgn1 in autism-related behaviors (Nguyen et al., 2020), we now offer genetic and pharmacological avenues to inhibit *Nlgn1* for modulating TSC-related phenotypes (Figure 2; Figure 3; Figure 4). Neuroligins are cell-adhesion molecules critical for synaptic structure function and the regulation of the E/I balance; *Nlgn1* is predominantly expressed in excitatory synapses (Liu et al., 2022). Thus, correcting *Nlgn1* protein expression in *Tsc2* ^*+/−*^ mice may engender synaptic remodeling and correction of behaviors by normalizing excitatory transmission. Compared to our previous strategy employing lentiviruses expressing shRNA against *Nlgn1*, we now provide genetic evidence that we can achieve rescue by inducing *Nlgn1* haploinsufficiency in *Tsc2* ^*+/−*^ mice (Figure 2; Figure 3; Figure 4), which further supports a TSC-Nlgn1 axis in TSC pathology. Furthermore, 4EGI-1, an inhibitor of cap-dependent translation was successfully used to achieve the same objective. 4EGI-1 and other similar acting compounds, alone or in synergy with other kinase inhibitors, are being explored as cancer therapeutics and thus could be repurposed for neurodevelopmental disorders such as TSC (Mahalingam et al., 2014).

By correcting *Nlgn1* expression, we reversed the impairment of mGluR-LTD in *Tsc2* ^*+/−*^ mice. Previous work in mouse models of ASD (knockout models of *Shank3, Pten, Fmr1, Tsc, Cntnap2*, where mTORC1 signaling is dysregulated) has shown that mGluR-LTD is disrupted in various brain regions, including the prefrontal cortex and hippocampus (Lee et al., 2019). Moreover, mutations in genes related to mGluR signaling have been identified in individuals with ASD, further supporting a role for this pathway in the disorder (Hadley et al., 2014). mTORC1 inhibition with rapamycin rescued several behavioral phenotypes in *Tsc2* ^*+/−*^ mice (Ehninger and Silva, 2011) and in other models of neurodevelopmental disorders (Girodengo et al., 2022). Strikingly, we achieved rescue of TSC-related phenotypes in the *Tsc2* ^*+/−*^ mice, without correcting hyperactivated mTORC1 (Figure 4). Both *Nlgn1* deletion and 4EGI-1 treatments did not alter rpS6 phosphorylation in animals where cellular (mGluR-LTD) and behavioral (context discrimination and social behavior) were normalized. This is an intriguing finding, suggesting that targeting mTORC1 downstream molecules, such as *Nlgn1*, in complex regulatory pathways is an appealing strategy, which may negate off-target effects and offer targeted therapeutics based on mechanistic understanding. In addition to mRNA translation, mTORC1 regulates several processes such as lipid metabolism and autophagy and thus our findings highlight the important role of translational control, downstream of mTORC1, in TSC.

### 4.3 Study limitations and perspective—relevance to treatment

We focused on the mTORC1/4E-BP/Nlgn1 axis and did not perform an exhaustive analysis of other candidate signaling pathways, such as ERK, which may be corrected in *Tsc2* ^*+/−*^ brains following normalization of *Nlgn1* protein amounts. Moreover, we focused on hippocampus only and did not analyze other brain areas relevant to ASD/TSC such as forebrain or cerebellum. Further elucidation of the synaptic role of *Nlgn1* and possibly other neuroligins in TSC and other forms of ASD is required. For example, examining other forms of synaptic plasticity, apart from LTD in ASD/TSC models may reveal new pharmacological avenues for treatment. Finally, our experiments were performed on adult mice. Potential clinical application of the mechanistic findings of this study in early life would require the study of younger animals.

Overall, our study revealed that reducing *Nlgn1* expression is a possible new therapeutic avenue for TSC and plausibly for other neurodevelopmental disorders.

## Data Availability

The raw data supporting the conclusion of this article will be made available by the authors, without undue reservation.

## References

[B1] Auerbach B. D. Osterweil E. K. Bear M. F. (2011). Mutations causing syndromic autism define an axis of synaptic pathophysiology. Nature 480, 63–68. 10.1038/nature10658 22113615 PMC3228874

[B2] Blundell J. Blaiss C. A. Etherton M. R. Espinosa F. Tabuchi K. Walz C. (2010). Neuroligin-1 deletion results in impaired spatial memory and increased repetitive behavior. J. Neurosci. 30, 2115–2129. 10.1523/JNEUROSCI.4517-09.2010 20147539 PMC2824441

[B3] Chen Y. C. Chang Y. W. Huang Y. S. (2019). Dysregulated translation in neurodevelopmental disorders: An overview of autism-risk genes involved in translation. Dev. Neurobiol. 79, 60–74. 10.1002/dneu.22653 30430754

[B4] Chu J. Pelletier J. (2018). Therapeutic opportunities in eukaryotic translation. Cold Spring Harb. Perspect. Biol. 10, a032995. 10.1101/cshperspect.a032995 29440069 PMC5983196

[B5] Dahlhaus R. Hines R. M. Eadie B. D. Kannangara T. S. Hines D. J. Brown C. E. (2010). Overexpression of the cell adhesion protein neuroligin-1 induces learning deficits and impairs synaptic plasticity by altering the ratio of excitation to inhibition in the hippocampus. Hippocampus 20, 305–322. 10.1002/hipo.20630 19437420

[B6] Doernberg E. Hollander E. (2016). Neurodevelopmental disorders (ASD and ADHD): DSM-5, ICD-10, and ICD-11. CNS Spectr. 21, 295–299. 10.1017/S1092852916000262 27364515

[B7] Ehninger D. Han S. Shilyansky C. Zhou Y. Li W. Kwiatkowski D. J. (2008). Reversal of learning deficits in a Tsc2+/- mouse model of tuberous sclerosis. Nat. Med. 14, 843–848. 10.1038/nm1788 18568033 PMC2664098

[B8] Ehninger D. Silva A. J. (2011). Rapamycin for treating Tuberous sclerosis and Autism spectrum disorders. Trends Mol. Med. 17, 78–87. 10.1016/j.molmed.2010.10.002 21115397 PMC3075964

[B9] Faul F. Erdfelder E. Buchner A. Lang A. G. (2009). Statistical power analyses using G*power 3.1: Tests for correlation and regression analyses. Behav. Res. Methods 41, 1149–1160. 10.3758/BRM.41.4.1149 19897823

[B10] Garami A. Zwartkruis F. J. Nobukuni T. Joaquin M. Roccio M. Stocker H. (2003). Insulin activation of Rheb, a mediator of mTOR/S6K/4E-BP signaling, is inhibited by TSC1 and 2. Mol. Cell 11, 1457–1466. 10.1016/s1097-2765(03)00220-x 12820960

[B11] Girodengo M. Ultanir S. K. Bateman J. M. (2022). Mechanistic target of rapamycin signaling in human nervous system development and disease. Front. Mol. Neurosci. 15, 1005631. 10.3389/fnmol.2022.1005631 36226315 PMC9549271

[B12] Gkogkas C. G. Khoutorsky A. Ran I. Rampakakis E. Nevarko T. Weatherill D. B. (2013). Autism-related deficits via dysregulated eIF4E-dependent translational control. Nature 493, 371–377. 10.1038/nature11628 23172145 PMC4133997

[B13] Hadley D. Wu Z. L. Kao C. Kini A. Mohamed-Hadley A. Thomas K. (2014). The impact of the metabotropic glutamate receptor and other gene family interaction networks on autism. Nat. Commun. 5, 4074. 10.1038/ncomms5074 24927284 PMC4059929

[B14] Hinnebusch A. G. Ivanov I. P. Sonenberg N. (2016). Translational control by 5'-untranslated regions of eukaryotic mRNAs. Science 352, 1413–1416. 10.1126/science.aad9868 27313038 PMC7422601

[B15] Hooshmandi M. Wong C. Khoutorsky A. (2020). Dysregulation of translational control signaling in autism spectrum disorders. Cell Signal 75, 109746. 10.1016/j.cellsig.2020.109746 32858122

[B16] Ingolia N. T. Ghaemmaghami S. Newman J. R. Weissman J. S. (2009). Genome-wide analysis *in vivo* of translation with nucleotide resolution using ribosome profiling. Science 324, 218–223. 10.1126/science.1168978 19213877 PMC2746483

[B17] Kelleher R. J. Bear M. F. (2008). The autistic neuron: Troubled translation? Cell 135, 401–406. 10.1016/j.cell.2008.10.017 18984149

[B18] Lee K. Vyas Y. Garner C. C. Montgomery J. M. (2019). Autism-associated Shank3 mutations alter mGluR expression and mGluR-dependent but not NMDA receptor-dependent long-term depression. Synapse 73, e22097. 10.1002/syn.22097 30868621

[B19] Liu G. Y. Sabatini D. M. (2020). mTOR at the nexus of nutrition, growth, ageing and disease. Nat. Rev. Mol. Cell Biol. 21, 183–203. 10.1038/s41580-019-0199-y 31937935 PMC7102936

[B20] Liu X. Hua F. Yang D. Lin Y. Zhang L. Ying J. (2022). Roles of neuroligins in central nervous system development: Focus on glial neuroligins and neuron neuroligins. J. Transl. Med. 20, 418. 10.1186/s12967-022-03625-y 36088343 PMC9463862

[B21] Louros S. R. Osterweil E. K. (2016). Perturbed proteostasis in autism spectrum disorders. J. Neurochem. 139, 1081–1092. 10.1111/jnc.13723 27365114 PMC5215415

[B22] Mahalingam P. Takrouri K. Chen T. Sahoo R. Papadopoulos E. Chen L. (2014). Synthesis of rigidified eIF4E/eIF4G inhibitor-1 (4EGI-1) mimetic and their *in vitro* characterization as inhibitors of protein-protein interaction. J. Med. Chem. 57, 5094–5111. 10.1021/jm401733v 24827861 PMC4216204

[B23] Mamane Y. Petroulakis E. Lebacquer O. Sonenberg N. (2006). mTOR, translation initiation and cancer. Oncogene 25, 6416–6422. 10.1038/sj.onc.1209888 17041626

[B24] Nguyen T. A. Lehr A. W. Roche K. W. (2020). Neuroligins and neurodevelopmental disorders: X-linked genetics. Front. Synaptic Neurosci. 12, 33. 10.3389/fnsyn.2020.00033 32848696 PMC7431521

[B25] Rosina E. Battan B. Siracusano M. Di Criscio L. Hollis F. Pacini L. (2019). Disruption of mTOR and MAPK pathways correlates with severity in idiopathic autism. Transl. Psychiatry 9, 50. 10.1038/s41398-018-0335-z 30705255 PMC6355879

[B26] Santini E. Huynh T. N. Macaskill A. F. Carter A. G. Pierre P. Ruggero D. (2013). Exaggerated translation causes synaptic and behavioural aberrations associated with autism. Nature 493, 411–415. 10.1038/nature11782 23263185 PMC3548017

[B27] Sato A. Kasai S. Kobayashi T. Takamatsu Y. Hino O. Ikeda K. (2012). Rapamycin reverses impaired social interaction in mouse models of tuberous sclerosis complex. Nat. Commun. 3, 1292. 10.1038/ncomms2295 23250422 PMC3535343

[B28] Simbriger K. Amorim I. S. Chalkiadaki K. Lach G. Jafarnejad S. M. Khoutorsky A. (2020). Monitoring translation in synaptic fractions using a ribosome profiling strategy. J. Neurosci. Methods 329, 108456. 10.1016/j.jneumeth.2019.108456 31610213 PMC6899497

[B29] Sonenberg N. (2008). eIF4E, the mRNA cap-binding protein: from basic discovery to translational research. Biochem. Cell Biol. 86, 178–183. 10.1139/O08-034 18443631

[B30] Touraine R. Hauet Q. Harzallah I. Baruteau A. E. (2022). Tuberous sclerosis complex: Genetic counselling and perinatal follow-up. Arch. Pediatr. 29, 5S3–5S7. 10.1016/S0929-693X(22)00283-4 36585068

[B31] Trobiani L. Meringolo M. Diamanti T. Bourne Y. Marchot P. Martella G. (2020). The neuroligins and the synaptic pathway in Autism Spectrum Disorder. Neurosci. Biobehav Rev. 119, 37–51. 10.1016/j.neubiorev.2020.09.017 32991906

[B32] Tuttle A. H. Tansley S. Dossett K. Tohyama S. Khoutorsky A. Maldonado-Bouchard S. (2017). Social propinquity in rodents as measured by tube cooccupancy differs between inbred and outbred genotypes. Proc. Natl. Acad. Sci. U. S. A. 114, 5515–5520. 10.1073/pnas.1703477114 28484016 PMC5448193

[B33] Vorstman J. A. S. Parr J. R. Moreno-De-Luca D. Anney R. J. L. Nurnberger J. I. Hallmayer J. F. (2017). Autism genetics: Opportunities and challenges for clinical translation. Nat. Rev. Genet. 18, 362–376. 10.1038/nrg.2017.4 28260791

