# Supplementary material for: Reversal of memory and autism-related phenotypes in Tsc2 +/− mice via inhibition of Nlgn1
Source: Front Cell Dev Biol. 2023 May 24;11:1205112. doi: 10.3389/fcell.2023.1205112 (PMC10244498; doi:10.3389/fcell.2023.1205112)
Supplement: Supplementary file 1 [file Table1.DOCX]

**Supplementary Table 1** Antibodies used for immunoblotting and immunofluorescence.

| **Antibody** | **Company** | **Cat. Number** | **Concentration** |
| --- | --- | --- | --- |
| Ribosomal protein S6 | Santa Cruz | sc-74459 | 1:5000 |
| Phospho- Ribosomal protein S6 | Cell Signaling | 2215 | 1:1000 |
| Tsc2 | Santa Cruz | sc-893 | 1:1000 |
| Nlgn1 | Synaptic Systems | 129013 | 1:1000 |
| Nlgn1 | Synaptic Systems | 129211 | 1:300 |
| Anti-mouse - HRP | Thermo-Fischer | 31430 | 1:5000 |
| Anti-rabbit - HRP | Thermo-Fischer | 31460 | 1:5000 |
| Anti-goat - HRP | Santa Cruz | sc-2020 | 1:5000 |
