# Supplementary material for: Reversal of memory and autism-related phenotypes in Tsc2 +/− mice via inhibition of Nlgn1
Source: Front Cell Dev Biol. 2023 May 24;11:1205112. doi: 10.3389/fcell.2023.1205112 (PMC10244498; doi:10.3389/fcell.2023.1205112)
Supplement: Supplementary file 2 [file Table2.DOCX]

**Supplementary Table 2** Statistical analysis details

| **Test** | **Mean ± S.E.M** | **Standard Deviation (SD)** | **Significance and multiple comparisons** | **Normality – Shapiro Wilk (α=0.05) Normal distribution (Gaussian) (Y/N); p-value** | **Parameter** | **N** | **Descriptive**  **Statistics** | **Figure** |
| --- | --- | --- | --- | --- | --- | --- | --- | --- |
| Students *t-*test | WT: 3.012 ± 0.194  *Tsc2^+/-^*: 3.099 ± 0.171 | WT: 0.336  *Tsc2^+/-^*: 0.296 | WT vs *Tsc2^+/-^*; p = 0.15 | WT Y;p=0.091  Tsc2+/- Y;p=0.133 | Absorbance (254 nm) | WT (3)  *Tsc2^+/-^* (3) | two-tailed, t=2.322, df=4 | **Fig. 1A** |
| Student’s *t-*test | ***Nlgn1***  WT: 0.727 ±0.029  *Tsc2^+/-^*: 1.344 ±0.121  ***Actb***  WT: 1.512 ±0.089  *Tsc2^+/-^*: 1.496 ±0.117 | ***Nlgn1***  WT: 0.050  *Tsc2^+/-^*: 0.210  ***Actb***  WT: 0.154  *Tsc2^+/-^*: 0.203 | WT vs *Tsc2^+/-^*; p = 0.003 | *Nlgn1*  WT Y;p=0.868  Tsc2+/- Y;p=0.231  *Actb*  WT Y;p=0.985  Tsc2+/- Y;p=0.411 | *Nlgn1* or *Actb* mRNA expression in heavy/light polysomes ratio (arbitrary units) | WT (3)  *Tsc2^+/-^* (3) | two-tailed, t=1.899, df=4 | **Fig. 1B** |
| Unpaired *t-*test | WT: 1.030± 0.020  *Tsc2^+/-^*: 1.430 ± 0.086 | WT: 0.040  *Tsc2^+/-^*: 0.172 | WT vs *Tsc2^+/-^*; p = 0.004 | WT Y;p=0.806  Tsc2+/- Y;p=0.979 | Nlgn1 protein expression (arbitrary units) | WT (4)  *Tsc2^+/-^* (4) | two-tailed, t=4.524, df=6 | **Fig. 1C** |
| Unpaired *t-*test | WT: 0.013± 0.003  *Tsc2^+/-^*: 0.028 ± 0.017 | WT: 0.006  *Tsc2^+/-^*: 0.034 | WT vs *Tsc2^+/-^*; p = 0.019 | WT Y;p=0.888  Tsc2+/- T;p=0.885 | Nlgn1 immunofluorescent intensity (arbitrary units) | WT (4)  *Tsc2^+/-^* (4) | two-tailed, t=3.163, df=6 | **Fig. 1D** |
| Unpaired *t-*test | ***Nlgn1***  WT: 0.809 ±0.0193  *Tsc2^+/-^*: 0.786 ±0.040  ***Actb***  WT: 1.144 ±0.063  *Tsc2^+/-^*: 1.133 ±0.097 | ***Nlgn1***  WT: 0.039  *Tsc2^+/-^*: 0.080  ***Actb***  WT: 0.126  *Tsc2^+/-^*: 0.194 | ***Nlgn1***  WT vs *Tsc2^+/-^*; p = 0.617  ***Actb***  WT vs *Tsc2^+/-^*; p = 0.897 | *Nlgn1*  WT Y;p=0.772  Tsc2+/- Y;p=0.913  *Actb*  WT Y;p=0.061  Tsc2+/- Y;p=0.128 | *Nlgn1* or *Actb* mRNA expression (arbitrary units) | WT (4)  *Tsc2^+/-^* (4) | *Nlgn1*: two-tailed, t=0.526, df=6  *Actb*  two-tailed, t=0.134, df=6 | **Fig. 1E** |
| One-way ANOVA with Bonferroni’s post-hoc | WT +vehicle: 1.075 ±0.022  *Tsc2^+/-^* +vehicle: 1.518 ±0.064  WT +rapamycin: 1.075 ±0.022  *Tsc2^+/-^* +rapamycin: 1.030 ±0.015  WT +4EGI-1: 1.135 ±0.075  *Tsc2^+/-^* +4EGI-1: 1.128 ±0.044 | WT +vehicle: 0.044  *Tsc2^+/-^* +vehicle: 0.128  WT +rapamycin: 0.044  *Tsc2^+/-^* +rapamycin: 0.030  WT +4EGI-1: 0.150  *Tsc2^+/-^* +4EGI-1: 0.088 | ANOVA p=0.0002  WT vehicle vs. *Tsc2^+/-^* vehicle p= 0.0004  WT vehicle vs. WT rapamycin p= 0.9927  WT vehicle vs. *Tsc2^+/-^* rapamycin p= 0.8702  WT vehicle vs. WT 4EGI-1 p= 0.9738  WT vehicle vs. *Tsc2^+/-^* 4EGI-1 p= 0.9854  *Tsc2^+/-^* vehicle vs. WT rapamycin p= 0.0001  *Tsc2^+/-^* vehicle vs. *Tsc2^+/-^* rapamycin p= 0.0044  *Tsc2^+/-^* vehicle vs. WT 4EGI-1 p= 0.0020  *Tsc2^+/-^* vehicle vs. *Tsc2^+/-^* 4EGI-1 p= 0.0016  WT rapamycin vs. *Tsc2^+/-^* rapamycin p= 0.5680  WT rapamycin vs. WT 4EGI-1 p= 0.7828  WT rapamycin vs. *Tsc2^+/-^* 4EGI-1 p= 0.8290  *Tsc2^+/-^* rapamycin vs. WT 4EGI-1 p= 0.9989  *Tsc2^+/-^* rapamycin vs. *Tsc2^+/-^* 4EGI-1 p= 0.9969  WT 4EGI-1 vs. *Tsc2^+/-^* 4EGI-1 p >0.9999 | WT +vehicle Y;p=0.851  *Tsc2^+/-^* +vehicle Y;p=0.952  WT +rapamycin Y;p=0.968  *Tsc2^+/-^* +rapamycin Y;p=0.798  WT +4EGI-1 Y;p=0.884  *Tsc2^+/-^* +4EGI-1 Y;p=0.915 | Nlgn1 protein expression | WT +vehicle (4)  *Tsc2^+/-^* +vehicle (4)  WT +rapamycin (4)  *Tsc2^+/-^* +rapamycin (4)  WT +4EGI-1 (4)  *Tsc2^+/-^* +4EGI-1 (4) | F (5, 18) = 9.298 | **Fig. 1F** |
| One-way ANOVA with Bonferroni’s post-hoc | WT: 1.023 ±0.037  *Tsc2^+/-^*: 1.493 ±0.081  *Nlgn1^+/-^*: 0.840 ±0.074  *Tsc2^+/-^/Nlgn1^+/-^*: 1.038 ±0.049 | WT: 0.074  *Tsc2^+/-^*: 0.162  *Nlgn1^+/-^*: 0.148  *Tsc2^+/-^/Nlgn1^+/-^*: 0.098 | WT vs. *Tsc2^+/-^* p=0.001  WT vs. *Nlgn1^+/-^* p=0.227  WT vs. Tsc2^+/-^/*Nlgn1^+/-^* p<0.0001  Tsc2+/- vs. *Nlgn1^+/-^* p=0.001  Tsc2+/- vs. *Tsc2^+/-^/Nlgn1^+/-^* p=0.001  Nlgn1+/- vs. *Tsc2^+/-^/Nlgn1^+/^* p=0.176 | WT Y;p=0.981  *Tsc2^+/-^* Y;p=0.941  *Nlgn1^+/-^* Y;p=0.963  *Tsc2^+/-^/Nlgn1^+/-^* Y;p=0.877 | Nlgn1 protein expression | WT (4)  *Tsc2^+/-^* (4)  *Nlgn1^+/-^* (4)  *Tsc2^+/-^/Nlgn1^+/-^* (4) | F (3, 12) = 19.27 | **Fig. 2B** |
| One-way ANOVA with Bonferroni’s post-hoc | WT: 41.176 ±5.800  *Tsc2^+/-^* : 18.616 ±5.750  *Nlgn1^+/-^*: 35.531 ±5.088  *Tsc2^+/-^/Nlgn1^+/-^*: 43.943 ±4.612 | WT: 20.912  *Tsc2^+/-^* : 19.919  *Nlgn1^+/-^*: 15.264  *Tsc2^+/-^/Nlgn1^+/-^*: 18.448 | WT vs. *Tsc2^+/-^* p=0.0237  WT vs. *Nlgn1^+/-^* p=0.902  WT vs. *Tsc2^+/-^/Nlgn1^+/-^* p= 0.979  Tsc2+/- vs. *Nlgn1^+/-^* p=0.1955  *Tsc2^+/-^* vs. *Tsc2^+/-^/Nlgn1^+/-^* p=0.0057  *Nlgn1^+/-^* vs. *Tsc2^+/-^/Nlgn1^+/^* p=0.7133 | WT Y;p=0.821  *Tsc2^+/-^* Y;p=0.743  *Nlgn1^+/-^* Y;p=0.162  *Tsc2^+/-^/Nlgn1^+/-^* Y;p=0.193 | % of LTD | WT (13)  *Tsc2^+/-^* (12)  *Nlgn1^+/-^* (9)  *Tsc2^+/-^/Nlgn1^+/-^* (16) | F (3, 46) = 4.604 p=0.0067 | **Fig. 2C** |
| Two-way ANOVA with Bonferroni’s post-hoc | **Baseline**  WT: 8.200  ±1.280  *Tsc2^+/-^* : 9.400  ±1.6343  *Tsc2^+/-^/Nlgn1^+/-^*: 8.300  ±1.551  *Nlgn1^+/-^*: 9.400  ±1.318  **Familiar**  WT: 68.1 ±2.635  *Tsc2^+/-^* : 57.100 ±3.009  *Tsc2^+/-^/Nlgn1^+/-^*: 43.943 ±4.612  *Nlgn1^+/-^*: 61.200 ±2.360  **Novel**  WT: 29.300 ±2.022  *Tsc2^+/-^* : 63.300 ±2.525  *Tsc2^+/-^/Nlgn1^+/-^*: 46.700 ±4.917  *Nlgn1^+/-^*: 31.600 ±2.093 | **Baseline**  WT: 4.048  *Tsc2^+/-^* : 5.167  *Tsc2^+/-^/Nlgn1^+/-^*: 4.905  *Nlgn1^+/-^*: 4.168  **Familiar**  WT: 8.333  *Tsc2^+/-^* : 9.515  *Tsc2^+/-^/Nlgn1^+/-^*: 14.584  *Nlgn1^+/-^*: 7.463  **Novel**  WT: 3.232  *Tsc2^+/-^* : 7.985  *Tsc2^+/-^/Nlgn1^+/-^*: 15.549  *Nlgn1^+/-^*: 6.619 | **WT**  baseline vs. familiar p<0.001  baseline vs. novel p<0.001  familiar vs. novel p<0.001  ***Tsc2^+/-^***  baseline vs. familiar p<0.001  baseline vs. novel p<0.001  familiar vs. novel p>0.999  ***Tsc2^+/-^/Nlgn1^+/-^***  baseline vs. familiar p<0.001  baseline vs. novel p<0.001  familiar vs. novel p=0.01  ***Nlgn1^+/-^***  baseline vs. familiar p<0.001  baseline vs. novel p<0.001  familiar vs. novel p<0.001 | **Baseline**  Y;p=0.764  **Familiar**  Y;p=0.973  **Novel**  Y;p=0.901 | % Freezing | WT (10)  *Tsc2^+/-^* (10)  *Tsc2^+/-^/Nlgn1^+/-^* (10)  *Nlgn1^+/-^* (10) | Genotype  F (3, 108) = 12.06 p<0.001  Context  F (2, 108) = 481.3 p<0.001  Genotype x Context  F (6, 108) = 16.13 p<0.001 | **Fig. 3A** |
| Two-way ANOVA with Bonferroni’s post-hoc | **Baseline**  WT +veh.: 9.200±1.041  *Tsc2^+/-^* +veh.: 8.800±1.143  WT +4EGI-1: 8.000±0.919  *Tsc2^+/-^* +4EGI-1: 9.600±1.067  **Familiar**  WT +veh.: 60.900±1.760  *Tsc2^+/-^* +veh.:63.200 ±2.407  WT +4EGI-1: 58.300±3.144  *Tsc2^+/-^* +4EGI-1:61.700 ±3.496  **Novel**  WT +veh.: 32.100±2.869  *Tsc2^+/-^* +veh.: 59.100±3.516  WT +4EGI-1: 28.400±2.642  *Tsc2^+/-^* +4EGI-1: 50.300±2.890 | **Baseline**  WT +veh.: 3.292  *Tsc2^+/-^* +veh.: 3.614  WT +4EGI-1: 2.906  *Tsc2^+/-^* +4EGI-1: 3.374  **Familiar**  WT +veh.: 5.566  *Tsc2^+/-^* +veh.: 7.612  WT +4EGI-1: 9.942  *Tsc2^+/-^* +4EGI-1: 11.055  **Novel**  WT +veh.: 9.073  *Tsc2^+/-^* +veh.: 11.119  WT +4EGI-1:8.355  *Tsc2^+/-^* + 4EGI-1: 9.139 | **WT +veh.**  baseline vs. familiar p<0.001  baseline vs. novel p<0.001  familiar vs. novel p<0.001  ***Tsc2^+/-^* +veh**.  baseline vs. familiar p<0.001  baseline vs. novel p<0.001  familiar vs. novel p=0.710  **WT +4EGI-1**  baseline vs. familiar p<0.001  baseline vs. novel p<0.001  familiar vs. novel p<0.001  ***Tsc2^+/-^* +4EGI-1**  baseline vs. familiar p<0.001  baseline vs. novel p<0.001  familiar vs. novelp=0.003 | **Baseline**  Y;p=0.971  **Familiar**  Y;p=0.970  **Novel**  Y;p=0.904 | % Freezing | WT +veh. (10)  *Tsc2^+/-^* +veh. (10)  WT +4EGI-1 (10)  *Tsc2^+/-^* +4EGI-1 (10) | Genotype  F (3, 108) = 15.92  Context  F (2, 108) = 469.8  Genotype x Context  F (6, 108) = 10.47 | **Fig. 3B** |
| One-way ANOVA with Bonferroni’s post-hoc | WT: 110.2±6.796  *Nlgn1^+/-^:* 117.800±6.078  *Tsc2^+/-^:* 62.670±5.291  *Tsc2^+/-^/ Nlgn1^+/-^:* 109.300±6.589 | WT: 23.542  *Nlgn1^+/-^:* 21.055  *Tsc2^+/-^:* 18.329  *Tsc2^+/-^/ Nlgn1^+/-^:* 22.825 | WT vs. *Nlgn1^+/-^* p=0.823  WT vs. *Tsc2^+/-^* p<0.0001  WT vs. *Tsc2^+/-^/ Nlgn1^+/-^* p=0.999  *Nlgn1^+/-^* vs. *Tsc2^+/-^* p<0.0001  *Nlgn1^+/-^* vs. *Tsc2^+/-^/ Nlgn1^+/-^* p=0.774  *Tsc2^+/-^* vs. *Tsc2^+/-^/ Nlgn1^+/-^* p<0.0001 | WT Y;p=0.939  *Nlgn1^+/-^* Y;p=0.948  *Tsc2^+/-^* Y;p=0.934  *Tsc2^+/-^/ Nlgn1^+/-^* Y;p=0.947 | Active Interaction time (s) | WT (12)  *Nlgn1^+/-^* (12)  *Tsc2^+/-^* (12)  *Tsc2^+/-^/ Nlgn1^+/-^* (12) | F (3, 44) = 16.39 p<0.0001 | **Fig. 4B** |
| One-way ANOVA with Bonferroni’s post-hoc | WT +veh.: 96.60±5.779  *Tsc2^+/-^* +veh.: 60.500±5.379  WT +4EGI-1: 97.910±5.816  *Tsc2^+/-^* +4EGI-1: 104.600±7.752 | WT +veh.: 18.275  *Tsc2^+/-^* +veh.: 18.633  WT +4EGI-1: 19.289  *Tsc2^+/-^* +4EGI-1: 24.514 | WT vehicle vs. Tsc2+/- vehicle p=0.001  WT vehicle vs. WT 4EGI-1 p=0.998  WT vehicle vs. Tsc2+/- 4EGI-1 p=0.817  Tsc2+/- vehicle vs. WT 4EGI-1 p=0.0005  Tsc2+/- vehicle vs. Tsc2+/- 4EGI-1 p<0.0001  WT 4EGI-1 vs. Tsc2+/- 4EGI-1 p=0.876 | WT +veh. Y;p=0.973  *Tsc2^+/-^* +veh.: Y;p=0.966  WT +4EGI-1: Y;p=0.977  *Tsc2^+/-^* +4EGI-1: Y;p=0.981 | Active Interaction time (s) | WT +veh. (10)  *Tsc2^+/-^* +veh. (12)  WT +4EGI-1 (11)  *Tsc2^+/-^* +4EGI-1 (10) | F (3, 39) = 10.89 p<0.0001 | **Fig. 4C** |
| Two-way ANOVA with Bonferroni’s post-hoc | **Co-Occupancy**  WT: 86.083±2.050  *Tsc2^+/-^*: 29.083±0.793  *Tsc2^+/-^/* *Nlgn1^+/-^:* 84.417±2.497  *Nlgn1^+/-^:* 87.583±1.747  **Single Occupancy**  WT: 7.250±1.298  *Tsc2^+/-^*: 38.917±0.543  Tsc2+/-/ Nlgn1+/-: 9.250±1.441  *Nlgn1^+/-^:* 8.000±1.249  **Vacant**  WT: 6.667±1.755  *Tsc2^+/-^*: 32.000±1.015  Tsc2+/-/ Nlgn1+/-: 6.333±1.389  *Nlgn1^+/-^:* 4.417±0.892 | **Co-Occupancy**  WT: 7.101  *Tsc2^+/-^*: 3.440  Tsc2^+/-^/ Nlgn1^+/-^: 8.650  *Nlgn1^+/-^:* 6.052  **Single Occupancy**  WT: 4.496  *Tsc2^+/-^*: 1.881  Tsc2+/-/ Nlgn1+/-: 4.992  *Nlgn1^+/-^:* 4.327  **Vacant**  WT: 6.079  *Tsc2^+/-^*: 3.526  Tsc2+/-/ Nlgn1+/-: 4.812  *Nlgn1^+/-^:* 3.090 | **Co-Occupancy**  WT vs. *Tsc2^+/-^* p<0.0001  WT vs. *Tsc2^+/-^/Nlgn1^+/-^* p=0.858  WT vs. *Nlgn1^+/-^* p=0.891  *Tsc2^+/-^* vs. *Tsc2^+/-^/Nlgn1^+/-^* p<0.0001  *Tsc2^+/-^* vs. *Nlgn1^+/-^* p<0.0001  *Tsc2^+/-^/*Nlgn1^+/-^ vs. *Nlgn1^+/-^* p=0.437  **Single Occupancy**  WT vs. *Tsc2^+/-^* p<0.0001  WT vs. *Tsc2^+/-^/Nlgn1^+/-^* p=0.777  WT vs. *Nlgn1^+/-^* p=0.984  *Tsc2^+/-^* vs. *Tsc2^+/-^/Nlgn1^+/-^* p<0.0001  *Tsc2^+/-^* vs. *Nlgn1^+/-^* p<0.0001  *Tsc2^+/-^/*Nlgn1^+/-^ vs. *Nlgn1^+/-^* p=0.933  **Vacant**  WT vs. *Tsc2^+/-^* p<0.0001  WT vs. *Tsc2^+/-^/Nlgn1^+/-^* p=0.998  WT vs. *Nlgn1^+/-^* p=0.708  *Tsc2^+/-^* vs. *Tsc2^+/-^/Nlgn1^+/^* p<0.0001*^-^*  *Tsc2^+/-^* vs. *Nlgn1^+/-^* p<0.0001  *Tsc2^+/-^/*Nlgn1^+/-^ vs. *Nlgn1^+/-^* p=0.799 | **Co-Occupancy** Y;p=0.813  **Single Occupancy** Y;p=0.771  **Vacant** Y;p=0.923 | % Occupancy | WT (12)  *Nlgn1^+/-^* (12)  *Tsc2^+/-^* (12)  *Tsc2^+/-^/ Nlgn1^+/-^* (12) | Genotype  F (3, 132) = 892 p>0.9999  Occupancy  F (2, 132) = 209 p<0.0001  Genotype x Occupancy  F (6, 132) = 275.9 p<0.0001 | **Fig. 4E** |
| Two-way ANOVA with Bonferroni’s post-hoc | **Co-Occupancy**  WT: 86.33±1.432  *Tsc2^+/-^* +vehicle: 30.75±2.456  WT +vehicle: 83.91±2.094  *Tsc2^+/-^* +4EGI-1: 84.58±1.747  **Single Occupancy**  WT: 6.833±1.173  *Tsc2^+/-^* +vehicle: 33.083±1.734  WT +vehicle: 8.500±0.917  *Tsc2^+/-^* +4EGI-1 10.333±1.227  **Vacant**  WT: 6.833±1.381  *Tsc2^+/-^* +vehicle: 36.167±3.202  WT +vehicle: 7.583±1.564  *Tsc2^+/-^* +4EGI-1 5.083±0.763 | **Co-Occupancy**  WT: 4.961  *Tsc2^+/-^* +vehicle: 8.508  WT +vehicle: 7.254  *Tsc2^+/-^* +4EGI-1:v6.052  **Single Occupancy**  WT: 4.063  *Tsc2^+/-^* +vehicle: 6.007  WT +vehicle: 3.177  *Tsc2^+/-^* +4EGI-1: 4.250  **Vacant**  WT: 4.784  *Tsc2^+/-^* +vehicle: 11.092  WT +vehicle: 5.418  *Tsc2^+/-^* +4EGI-1: 2.643 | **WT vehicle**  Co-Occupancy vs. Single Occupancy p<0.0001  Co-Occupancy vs. Vacant p<0.0001  Single Occupancy vs. Vacant p>0.999  ***Tsc2^+/-^* vehicle**  Co-Occupancy vs. Single Occupancy p=0.619  Co-Occupancy vs. Vacant p=0.080  Single Occupancy vs. Vacant p=0.435  **WT 4EGI-1**  Co-Occupancy vs. Single Occupancy p<0.0001  Co-Occupancy vs. Vacant p<0.0001  Single Occupancy vs.Vacant p=0.928  ***Tsc2^+/-^* 4EGI-1**  Co-Occupancy vs. Single Occupancy p<0.0001  Co-Occupancy vs. Vacant p<0.0001  Single Occupancy vs. Vacant p=0.093 | **Co-Occupancy** Y;p=0.911  **Single Occupancy** Y;p=0.881  **Vacant** Y;p=0.712 | %Occupancy | WT +veh. (12)  *Tsc2^+/-^* +veh. (12)  WT +4EGI-1 (12)  *Tsc2^+/-^* +4EGI-1 (12) | Genotype  F (3, 132) = 1000 p>0.9999  Occupancy  F (2, 132) = 1393 p<0.0001  Genotype x Occupancy  F (6, 132) = 177.7 p<0.0001 | **Fig. 4F** |
| One-way ANOVA with Bonferroni’s post-hoc | WT: 1.035±0.025  *Tsc2^+/-^*: 1.540±0.109  *Tsc2^+/-^/ Nlgn1^+/-^*:1.523±0.120  *Nlgn1^+/-^*:1.033±0.041 | WT: 0.050  *Tsc2^+/-^*: 0.218  *Tsc2^+/-^/ Nlgn1+/-:* 0.240  *Nlgn1^+/-^*: 0.082 | WT vs. *Tsc2^+/-^* p=0.005  WT vs. *Tsc2^+/-^ Nlgn1^+/-^* p>0.999  WT vs. *Nlgn1^+/-^* p=0.998  *Tsc2^+/-^* vs. *Tsc2^+/-^ Nlgn1^+/-^*  *Tsc2^+/-^* vs. *Nlgn1^+/-^* p=0.005  *Tsc2^+/-^* /*Nlgn1^+/-^* vs. *Nlgn1^+/-^* p=0.007 | WT Y;p=0.980  *Tsc2^+/-^* Y;p=0.941  *Tsc2^+/-^/ Nlgn1^+/-^* Y;p=0.963  *Nlgn1^+/-^* Y;p=0.877 | Phopsho-S6 expression | WT (4)  *Tsc2^+/-^* (4)  *Tsc2^+/-^/ Nlgn1^+/-^* (4)  *Nlgn1^+/-^* (4) | F (3, 12) = 11.37 p=0.0008 | **Fig. 4G** |
| One-way ANOVA with Bonferroni’s post-hoc | WT +veh.: 1.020±0.031  *Tsc2^+/-^* +veh.: 1.365±0.094  WT +4EGI-1: 1.023±0.033  *Tsc2^+/-^* +4EGI-1: 1.375±0.085 | WT +veh.: 0.062  *Tsc2^+/-^* +veh.: 0.188  WT +4EGI-1: 1.023±0.066  *Tsc2^+/-^* +4EGI-1: 0.170 | WT vehicle vs. *Tsc2^+/-^* vehicle p=0.016  WT vehicle vs. WT 4EGI-1 p>0.999  WT vehicle vs. *Tsc2+/-* 4EGI-1 p=0.0140  *Tsc2^+/-^* vehicle vs. WT 4EGI-1 p=0.017  *Tsc2^+/-^* vehicle vs. *Tsc2^+/-^* 4EGI-1 p=0.999  WT 4EGI-1 vs. *Tsc2^+/-^* 4EGI-1 p=0.014 | WT +veh. Y; p=0.864  *Tsc2^+/-^* +veh. Y; p=0.931  WT +4EGI-1 Y; p=0.934  *Tsc2^+/-^* +4EGI-1 Y; p=0.942 | Phospho-S6 expression | WT +veh. (4)  *Tsc2^+/-^* +veh. (4)  WT +4EGI-1 (4)  *Tsc2^+/-^* +4EGI-1 (4) | F (3, 12) = 8.798 p=0.0023 | **Fig. 4H** |
